# Supplementary material for: Estimating COVID-19 vaccine uptake and its drivers among migrants, homeless and precariously housed people in France
Source: Commun Med (Lond). 2023 Feb 20;3:30. doi: 10.1038/s43856-023-00257-1 (PMC9939372; doi:10.1038/s43856-023-00257-1)
Supplement: Supplementary file 1 — Supplementary Information [file 43856_2023_257_MOESM1_ESM.pdf]

# Supplementary Information

## Table of Contents


Supplementary Information ..... 1

Supplementary Notes 1. National Vaccination Strategy ..... 2

Supplementary Notes 2. Actors involved in vaccination activities among PEH/PH ..... 5

In Ile de France Region ..... 5

In Marseille Region ..... 6

Supplementary Notes 3. French system: Types of facilities for roofless, homeless and migrant populations ..... 7

Supplementary Methods 1. Sample Size Calculation and Sampling Frames ..... 9

Supplementary Methods 2. Statistical Analysis Plan ..... 11

Direct Standardization to compare vaccine uptake ..... 11

Missing values ..... 11

Univariate ..... 12

Multivariate ..... 12

Stratified analyses ..... 12

Site-level analyses ..... 12

Supplementary Figures ..... 14

Figure S1. European Typology of Homelessness and Housing Exclusion ..... 15

Figure S2. Maps of Recruitment Sites in Ile de France Region ..... 16

Figure S3. Map of Recruitment Sites in Marseille Metropolitan Area ..... 17

Figure S4. Flow between original strata and strata for analysis (alluvium) ..... 18

Figure S5. Vaccine Uptake per recruitment site (Weighted Proportions and 95%CI) ..... 19

Figure S6. Forest Plot for the Multivariate Negative Binomial Regression (Site level variables) .. 21

Supplementary References ..... 22

## Supplementary Notes 1. National Vaccination Strategy

Source Ministry of Health (in French) : https://solidarites-sante.gouv.fr/grands-dossiers/vaccin-covid-19/je-suis-un-particulier/article/foire-aux-questions-la-strategie-de-vaccination-et-le-calendrier )

OBJECTIVES AND PRINCIPLES OF THE VACCINATION STRATEGY

The vaccine strategy put in place follows three public health objectives:

- To reduce mortality and severe forms of the disease
- To protect caregivers and the health care system
- To guarantee the safety of vaccines and vaccination

The vaccination should be non-mandatory, free of charge and without requirements (medical coverage, citizenship etc) and must be the safest possible.

Vaccination Announcements Timeline

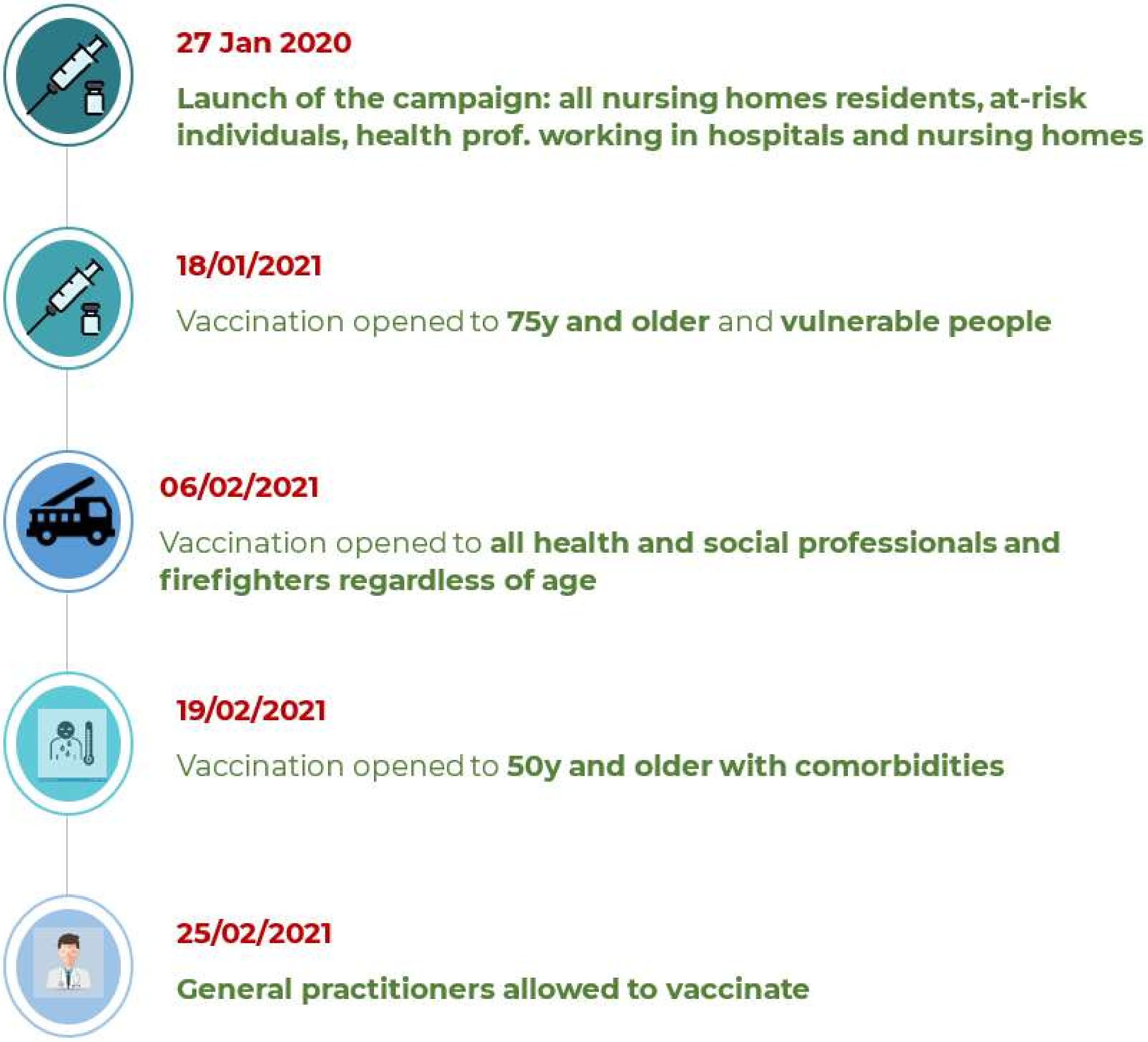

**15/03/2021**

**City drugstores and pharmacists allowed to vaccinate**

**08/04/2021**

Vaccination opened to **pregnant women (>= 2d trimester) regardless of age**

**12/04/2021**

Vaccination opened to **55y and older**

**01/05/2021**

Vaccination opened to **18y and older with comorbidities**

**12/05/2021**

Vaccination opened to **18y and older if doses are available in vaccination centers**

**31/05/2021**

Vaccination opened **to 18y and older**

**09/06/2021**

Introduction of **Pass Sanitaire for 18y and older to access hospitals and to travel**

**15/06/2021**

Vaccination opened to **12y and older**

**06/07/2021**

President Macron announcement:

**Mandatory Pass Sanitaire for 12y and older**

**Extension to leisure and cultural venues**

**Mandatory vaccination for health professionals**

**PCR and antigenic tests not free anymore for non-vaccinated**

**21/07/2021**

Pass Sanitaire **now mandatory to access bars, pubs, concert, and sport venues with a capacity >=50 000**

**09/08/2021**

Pass Sanitaire now mandatory to **access all leisure and cultural venues, to access large malls (>=20 000 $m^2$) and to visit hospitals and nursing homes**

**01/09/2021**

**Booster dose (Pfizer/Moderna only) for vulnerable people**

**30/09/2021**

**Pass Sanitaire extended to 12y and older**

**05/10/2021**

**Booster dose for health professionals**

**27/11/2021**

**Booster** dose extended **to the entire population aged 18y and older**

**15/12/2021**

Vaccination opened to **children aged 5 to 11 y with comorbidities or living with at-risk persons**

## Supplementary Notes 2. Actors involved in vaccination activities among PEH/PH

### In Ile de France Region

**International Non-Governmental Organizations (INGOs)**

**Croix Rouge Française** (in French : https://www.croix-rouge.fr/Actualite/COVID-19-vaccination/Covid-19-Ouverture-d-un-centre-de-vaccination-sur-le-Campus-de-la-Croix-Rouge-francaise-a-Montrouge-2534 )

Mobile Vaccination Teams / outreach activities: sensitization, possible vaccination during outreach

Vaccination centres: small, light and mobile structures that can be set up quickly anywhere: in health establishments, gymnasiums or other public places

Logistical support: provision of vehicles to rapidly handle deliveries of vaccine batches, materials, personal protective equipment (PPE)

**Médecins du Monde ( https://www.medecinsdumonde.org/ )**

Sensitization (on-site and during outreach activities):

Information Meetings

**Samu Social** (In French: https://www.siao.paris/actualites/tous-ensemble-mobilises-pour-vacciner-les-plus-precaires )

Sensitization (on-site and during outreach activities): https://www.samusocial.paris/le-115-oriente-vers-les-lieux-de-vaccination-contre-le-covid-19

On-site vaccination in Social Hostels managed by Samu Social

Vaccibus ( https://www.samusocial.paris/vaccibus-aller-vers-les-usagers-pour-faciliter-la-vaccination )

Vaccination centres

Support to local partners managing CHU/CHRS: sensitization and communication

115 HelpLine: orientation towards vaccination centers and general guidance

**Médecins Sans Frontières** (in French : https://www.msf.fr/actualites/covid-19-plus-de-5-000-personnes-en-grande-precarite-vaccinees-par-msf-en-ile-de-france-et-apres )

Vaccination in Food Distributions Sites (Restos du Coeur and Aurore)

Mobile Teams: in MWH and Emergency Shelters in Paris and suburbs

Sensitization (on-site and during outreach activities)

Vaccination centre: Paris headquarters transformed into a vaccination centre during summer

Set-up of a Hotline to manage vaccination appointments (2d dose) and provide general guidance

**Local organizations (support to homeless and migrants in Paris and suburbs)**

**Aurore**: partnerships with MSF and Samu Social to sensitize and refer homeless people to vaccination sites ( https://www.lestransmetteurs.org/post/vacciner-les-plus-exclus-contre-le-covid-19-avec-l-association-aurore )

**France Terre d'Asile:** sensitization during outreach activities

## In Marseille Region

**COREHSAN initiative**: collaboration between the Marseille Municipality and Ministry of Health teams, Médecins du Monde and several homeless organizations to provide testing, care and vaccination to migrants and homeless people in the urban city of Marseille (in French: https://www.hopital-europeen.fr/corhesan )

**ASSAB Network** (in French: https://projetassab.org/2021/12/21/vaccination-covid-19-modalites-dacces/ )*

Referral to a vaccination center: facilitate appointment booking by contacting the center concerned directly (7 vaccination centers in different districts of Marseille)

On-site vaccination activities (in shelters, food distribution sites, squats etc) on demand to the Corhesan team

Transport of people to a vaccination centre on demand to the Corhesan team

Referral to temporary vaccination sites.

## Supplementary Notes 3. French system: Types of facilities for roofless, homeless and migrant populations

Figure S1 summarizes the European Typology of Homelessness and Housing Exclusion (ETHOS typology) categories (source: https://www.feantsa.org/en/toolkit/2005/04/01/ethos-typology-on-homelessness-and-housing-exclusion ).

Following explanations are extracted from : Fondation Abbe Pierre· l'État du mal-logement en France 2021[1] and Homelessness Services in Europe - EOH Comparative Studies on Homelessness - FEANTSEA 2018 [2]

<u>Homelessness in France (**ETHOS Operational category**)</u>

Homeless and houseless people can switch from any facility to any other (considering their volatile and unstable situation) several times over a long period before "settling down", if they ever do.

-People living in the streets, metro stations, parking lots, under the bridges or in informal settlements/tents (roofless), can sometimes spend the night in overnight emergency shelters (Accueil de Nuit) and have a shower/meal in daily emergency centers (Accueil de Jour). Accueils de Jour/de Nuit are usually managed by social / charity organizations. (**ETHOS 1 and 2**)

-People living in make-shift housing / slums are often regrouped by community/country of origin (eg· Roma community) (**ETHOS 11**)

-People living in squats and unofficial community dwellings, often regrouped by community/country of origin (**ETHOS 8**)

- Migrants and homeless hosted in Emergency Shelters for Homeless People (CHU) and Centers for Social Re-Integration (CHRS) (**ETHOS 3**):

People in need of an emergency shelter can call the 115 Helpline and be referred to an overnight shelter or a CHU. CHU have on-site staffing which is present 24 hours a day, with a mix of private bedrooms and dormitories being used. Someone can remain resident until more permanent supportive housing can be found, usually a Social Hotel, a social/collective accommodation or a CHRS (see below).

CHRS: services are focused on families and individuals who are experiencing serious difficulties in socioeconomic integration, which can include, but is not restricted to homelessness. Women and children, vulnerable young people and ex-offenders who are on probation may also use these services. CHRS offers transitional housing with support services, some of which is tailored for people with specific needs with a broad trend towards offering single rooms, although not all services were described as following this practice. Typical stays in CHRS services were described as being around six months.

- People hosted in Social Hotels (**ETHOS 7** for transitional service): services managed by the Samu Social organization (Pole Delta). People oriented by the 115 HelpLine to an accommodation, usually a former hotel for tourists, dedicated to hosting people in very precarious situations.,)

- People hosted in collective accommodations managed by social, charity and/or religious organizations (**ETHOS 3 and 4**)

- Migrants and asylum seekers hosted in accommodation for immigrants (Dispositif National d'Accueil) (**ETHOS 5**) :

(a) Centres for asylum seekers (CADA); (b) Emergency accommodation for asylum seekers (HUDA, AT-SA, PRAHDA, CAO); (c) Reception and administrative status examination centres (CAES)·

- Undocumented migrants waiting for papers upon arrival on French soil or under deportation from France orders can be detained in retention centers (usually in airports) ·(CRA) (**ETHOS 9**)

- People living in Migrant Workers Hostels (**ETHOS 12 and 13**): initiated by the French state; typically hosting migrant workers from North and West Africa, but not only. Built during the 1950s, their original purpose was two-fold: as a mean of monitoring a suspect foreign male population at a time of decolonization and workers' struggles, and as a short-term housing solution for a supposedly temporary migrant labour force. MWH are often managed by state agencies, and sometimes private organizations. From a temporary solution, they became a permanent housing for thousands of first- and second-generation migrants.

From the book by Alistair Hunter "Journey's End? Old Age in France's Migrant Worker Hostels » [3]

*"Yet against all expectations the hostels continue to exist today, hosting an ageing cohort of men whose presence in France has proved far from temporary. The men themselves also defy expectations:* ***'geographically single', they did not bring their wives and children to France*** *while they were working,* ***yet at retirement they do not return definitively****. Instead* ***they constantly travel back-and-forth between their hostels in France and their families in places of origin****, even in quite advanced states of frailty and ill-health."*

*"Despite their lack of affective ties to France and their retention of ties to the place of origin, the men in this situation have by and large not returned on a definitive basis. As a result,* ***the 700-or-so hostels operating in France today constitute a predominantly middle-aged, if not elderly, environment.****"*

*"[..] a labour market rationale also dictated the location and functioning of* ***the hostels, which were often constructed close to local employers and industrial zones****. "*

*"Thirdly, the hostels were explicitly designed as temporary accommodation for temporary workers. As such,* ***the hostels were built quickly and cheaply, according to substandard norms of construction*** *and using materials which were not designed to be durable. The long hours which the men worked meant that* ***the hostel room was usually considered only ever as a place of sleep, needing only to fit a bed and precious little else****."*

Plan Grand Froid (https://www.gouvernement.fr/risques/grand-froid )

Additional emergency shelters are provided between 1st November and 31st March, which usually involves pressing disused public buildings into use on a temporary basis. An increasing reliance on hotels to provide emergency accommodation has been reported in recent years, reflecting growing pressure on these systems, particularly in and around Paris, organized around the 115-emergency helpline national system.

# Supplementary Methods 1. Sample Size Calculation and Sampling Frames

Table S1. Assumptions and parameters for sample size calculations

| | CFA/ES* Ile-De-France | Social Hostels Ile-De-France | MWH** Ile-De-France | Street/Camps /Squats Paris | COVID Homeless Cohort Marseille |
|---|---|---|---|---|---|
| Null Hypothesis (%) | 55 | 55 | 55 | 60 | 60 |
| Power (β, in %) | 80 | 80 | 80 | 80 | 80 |
| Type I error (α, in %) | 5 | 5 | 5 | 5 | 5 |
| Total population (sampling frame) | 45,000 | 32,000 | 23,000 | 5,000 | 1,200 |
| Accuracy (%) – Bilateral test | 5 | 5 | 5 | 5 | 5 |
| Design Effect* | 3 | 3 | 3 | 1 | 1 |
| **Total participants to include** | **1000** | **1000** | **1000** | **384** | **367** |
| Expected Non-Response/Refusals (%) | 20 | 20 | 20 | 33 | 33 |
| Number of clusters | 50 | 50 | 50 | 24 | 31 |

*CFA/ES : Centres for Asylum Seekers/Emergency Shelters for homeless

**MWH : Migrant Workers Hostels

COVIDHomeless Cohort : cohort of homeless and accomodated migrants followed up for 2 years in Marseille

Assumptions for null hypotheses were made according to published literature on vaccine hesitancy in general French population (*Schwarzinger et al.* [4]) and in PEH/PH (*Longchamps et al.* [5])

**Sampling Frames**

Sampling frames were usually obtained through partners and updated for each of the survey strata during the preparation phase between September and November 2021.

CFA/ES :

The complete list of facilities was constructed from the FINESS (Fichier national des établissements sanitaires et sociaux) national database of the Direction de la recherche, des études, de l'évaluation et des statistiques (DREES), which lists all health and social facilities, including Centres d'Hébergement d'Urgence (CHU), Centres d'Hébergement et de Réinsertion Sociale (CHRS), CPH, CADA/HUDA, and other CAO. This was corrected and amended by the APUR database, on which we relied in the framework of a partnership. We then contacted the main social organizations (France Terre d'Asile - FTDA, CRF, Aurore, etc.) and operators/landlords (Adoma/CDC-Habitat, Coallia and ADEF) to ensure that the list was up to date.

Social Hotels (Samu Social)

For this stratum, a partnership via a data transfer agreement was signed with the Samu Social de Paris. The SIAO 75 then sent us the complete and updated list of all the hotels, centers and residences managed by the Pôle 115 and the Pôle Habitat of Samu Social.

MWH

The complete list of MWHs was extracted from the FINESS database. More and more FTMs are being transformed into social residences, so it was important to check the accuracy of the data with the main social landlords (Coallia, ADEF and Adoma/CDC-Habitat).

Street/Camps/Slums/Squats/Subway

This stratum is by definition very heterogeneous and official data is sorely lacking.

The City of Paris, in partnership with APUR and a large number of social organizations, has been organizing a census overnight called « Solidarity Night » for several years, aiming to exhaustively count all the people living on the streets in Paris, along the Seine banks, ring road embankments and the Vincennes and Boulogne parks. The covered area is divided into 320 districts that 1900 volunteers walk through in one night to interview all the people they meet in the streets, parking lots, bus shelters, parks and gardens, train and subway stations. APUR graciously sent us the complete dataset of the last Solidarity Night (25 March 2021).

In parallel, data on informal campsites in Paris and along the ring road were provided by the Unité d'Assistance aux Sans-Abris (UASA) of the City of Paris. Data for the camps in the cities bordering Seine-Saint-Denis were provided by the France Terre d'Asile - FTDA association.

The Régie Autonome des Transport Parisien (RATP) conducts a daily census of homeless people living in the subway corridors and stations. Its social data collection team shared the latest data for the preparation of the survey, and was then a direct operational partner in the survey implementation in subway stations.

Finally, the Délégation Interministérielle à l'Hébergement et à l'Accès au Logement (DIHAL) has been in charge of slum clearance since 2018. In particular, it lists all the informal sites and slums in France, and compiles this information on a platform that can be accessed free of charge upon request. The list of all slums and informal settlements in Ile-de-France has thus been extracted during preparation phase.

Marseille

Prospective & Coopération and various partner organizations have been following a cohort of approximately 1,200 homeless, migrants and other highly precarious people in the municipality of Marseille since 2020. Epicentre randomly selected the sites beforehand. The random selection of participants was then organized by the local partner Prospective & Coopération following the same methodology as for the strata in IDF.

# Supplementary Methods 2. Statistical Analysis Plan

## Direct Standardization to compare vaccine uptake

First dose intake and vaccine coverage in our study population were compared to French general population. A weighted direct standardization by age categories has been performed (age cut-offs : 18, 25, 40, 55, 65, >65) and 95% CI were computed for overall study population and for each strata (according to Inskip et al. [6]). Reference data were downloaded from the Assurance Maladie website (link below). These data include vaccination and population indicators (Insee) for the French metropolitan adult population, over the entire period). Source: https://datavaccin-covid.ameli.fr/explore/dataset/donnees-vaccination-par-tranche-dage-type-de-vaccin-et-departement/table/?sort=departement_residence&q=date%3D2022-01-09+and+not+(departement_residence:%27999%27+OR+departement_residence:%27Tout+d%C3%A9partement%27)&refine.type_vaccin=Tout+vaccin&refine.classe_age=TOUT_AGE

Table S2. Population breakdown by Age, for the Ile-de-France region and for the study population (All stratum and by stratum)

| Age | France (million) | France (%) | All stratum (%) (n=100567) | Accomodation (%) (N=73159) | Homeless (Streets) (%) (N=4620) | Housing (%) (N=22788) |
|---|---|---|---|---|---|---|
| 18·25) | 5·3 | 10·3 | 8·3 | 9·3 | 14·6 | 3·9 |
| 25·40) | 11·7 | 22·8 | 47·0 | 51·8 | 43·8 | 32·1 |
| 40·55) | 12·6 | 24·6 | 25·6 | 25·6 | 28·4 | 25·3 |
| 55·65) | 8·2 | 16·0 | 10·6 | 8·9 | 9·8 | 16·1 |
| 65·Inf) | 13·4 | 26·2 | 8·4 | 4·4 | 3·4 | 22·5 |
| Total | 51·2 | | | | | |

Direct standardization was done using the “DSR” package in R : https://rdrr.io/cran/dsr/

# Missing values

For variables with a high number of missing values (>5%), missing values mechanism assumed to be MAR mechanism (missing at random) and verified. For each variable, several imputations methods were compared (multiple regression, random forest) and the one which gave the lowest error rate was retained. Only imputed variables with an error rate lower than 20% were used for the multivariable model. Imputation was done with the MICE package in R : https://cran.r-project.org/web/packages/mice/mice.pdf

## Univariate

Univariate logistic regression analysis explored vaccine uptake associated factors for all strata combined. A multilevel multivariable logistic regression model was constructed with random intercepts for specific recruitment sites to account for clustering and random effects on several variables after testing for validity (see below). We included variables that could explain differences in vaccine uptake proportions.

## Multivariate

Only variables with $p \leq 0{\cdot}2$ after univariate were retained in the full multivariate model. Multicollinearity was verified prior to model selection and variables with a Variance Inflation Factor (VIF, for continuous variables) > 5 or a generalized VIF (GVIF, for discrete variables) >2·5 were dropped (according to Midi et al. [7]). Random effects were tested on the full model and selected in order to minimize the second-order Akaike Information Criteria (AICc). After random effects selection, fixed effects were selected with a backwards procedure, minimizing AIC.

We also 'forced' a few factors that were not significant in univariate but were relevant for model adjustment (potential confounding factors or factors known to be linked with vaccine uptake in the literature). Moreover, some factors appeared too important/interesting to miss (opinions about vaccination, in particular).

Validation of the final model consisted in standardizing residuals analysis (overdispersion, distribution, outliers) and computing coefficients of determination (according to Menard et al. [8]).

## Stratified analyses

Descriptive, univariate and multilevel multivariable analyses were performed for each individual stratum, following global sample procedure.

Stratum final models may differ from the final global model, since the individuals characteristics can vary significantly.

## Site-level analyses

We performed an unweighted negative binomial regression on the total number of vaccinated individuals by site as a count outcome and site-related variables as covariables following aforementioned procedure. Questions were usually asked to site managers for Housed and Acommodated participants moslty. Streets individuals were then excluded de facto from the analyses.

Variables retained for descriptive, univariate and multivariable analyses were:

Stratum

Presence of social workers on site

Presence of health professionals on site

Awareness Raising/sensitization activities

Personalized support/mobilization activities

Actual vaccination activities on site

Distance to closest vaccination site targeting PEH

Distance to closest pharmacist/drugstore

Distance to closest vaccination center

## Supplementary Methods 3. Reproducibility of the tables figures and analyses

Final Study dataset is available in the folder “Final dataset”. It does not contain original individual information or sensitive data.

Labels are variable names are originally in French and are available in the “Labels and Data dictionary” folder. Refer to the “analyses.publi_v2” file for English translation of names and categories.

R scripts for analyses are available in the corresponding folder.

1. A guide for separate analyses is available in **“analyses@Menu”**

2. First run the initialization scripts -> **“0.1_init_v01” “0.2.1_import_ref_v02” and “analyses.fonctions_v02.R”**

3. To reproduce Tables, univariate and multivariable analyses -> **"analyses.publi_V01" "analyses.glmm_VF.fr"**

4. To reproduce Maps -> **"analyses.carto_V04"**

5. to reproduce Standardized Vaccination Curves -> **“analyses.CV_std_publi_V04”**

6. to reproduce Stratified Analyses -> **“analyses.glmm_strates_V01”, “analyses.glmm_strate1_V11”, “analyses.glmm_strate2_V04” and “analyses.glmm_strate3_V02”**

## Supplementary Figures

### Figure S1. European Typology of Homelessness and Housing Exclusion

(source: https://www.feantsa.org/en/toolkit/2005/04/01/ethos-typology-on-homelessness-and-housing-exclusion )

| Conceptual Category | | | OPERATIONAL CATEGORY | | LIVING SITUATION | GENERIC DEFINITION |
|---|---|---|---|---|---|---|
| Conceptual Category | ROOFLESS | 1 | People Living Rough | 1.1 | Public space or external space | Living in the streets or public spaces, without a shelter that can be defined as living quarters |
| | ROOFLESS | 2 | People in emergency accommodation | 2.1 | Night shelter | People with no usual place of residence who make use of overnight shelter, low threshold shelter |
| | HOUSELESS | 3 | People in accommodation for the homeless | 3.1 | Homeless hostel | Where the period of stay is intended to be short term |
| | | | | 3.2 | Temporary accommodation | |
| | | | | 3.3 | Transitional supported accommodation | |
| | | 4 | People in Women's Shelter | 4.1 | Women's shelter accommodation | Women accommodated to experience of domestic violence and where the period of stay is intended to be short term |
| | | 5 | People in accommodation for immigrants | 5.1 | Temporary accommodation/reception centres | Immigrants in reception or short term accommodation due to their immigrant status |
| | | | | 5.2 | Migrant workers accommodation | |
| | | 6 | People due to be released from institutions | 6.1 | Penal institutions | No housing available prior to release |
| | | | | 6.2 | Medical institutions (*) | Stay longer than needed due to lack of housing |
| | | | | 6.3 | Children's institutions/homes | No housing identified (e.g. by 18th birthday) |
| | | 7 | People receiving longer-term support (due to homelessness) | 7.1 | Residential care for older homeless people | Long stay accommodation with care for formerly homeless people (normally more than one year) |
| | | | | 7.2 | Supported accommodation for formerly homeless people | |
| | INSECURE | 8 | People living in insecure accommodation | 8.1 | Temporarily with family/friends | Living in conventional housing but not the usual place of residence due to lack of housing |
| | | | | 8.2 | No legal (sub)tenancy | Occupation of dwelling with no legal tenancy illegal occupation of a dwelling |
| | | | | 8.3 | Illegal occupation of land | Occupation of land with no legal rights |
| | | 9 | People living under threat of eviction | 9.1 | Legal orders enforced (rented) | Where orders for eviction are operative |
| | | | | 9.2 | Re-possession orders (owned) | Where mortagee has legal order to re-possess |
| | | 10 | People living under threat of violence | 10.1 | Police recorded incidents | Where police action is taken to ensure place of safety for victims of domestic violence |
| | INADEQUATE | 11 | People living in temporary/ non-conventional structures | 11.1 | Mobile homes | Not intended as place of usual residence |
| | | | | 11.2 | Non-conventional building | Makeshift shelter, shack or shanty |
| | | | | 11.3 | Temporary structure | Semi-permanent structure hut or cabin |
| | | 12 | People living in unfit housing | 12.1 | Occupied dwellings unfit for habitation | Defined as unfit for habitation by national legislation or building regulations |
| | | 13 | People living in extreme over-crowding | 13.1 | Highest national norm of overcrowding | Defined as exceeding national density standard for floor-space or uesable rooms |

Note: Short stay is defined as normally less than one year; Long stay in defined as more than one year.

## Figure S2. Maps of Recruitment Sites in Ile de France Region

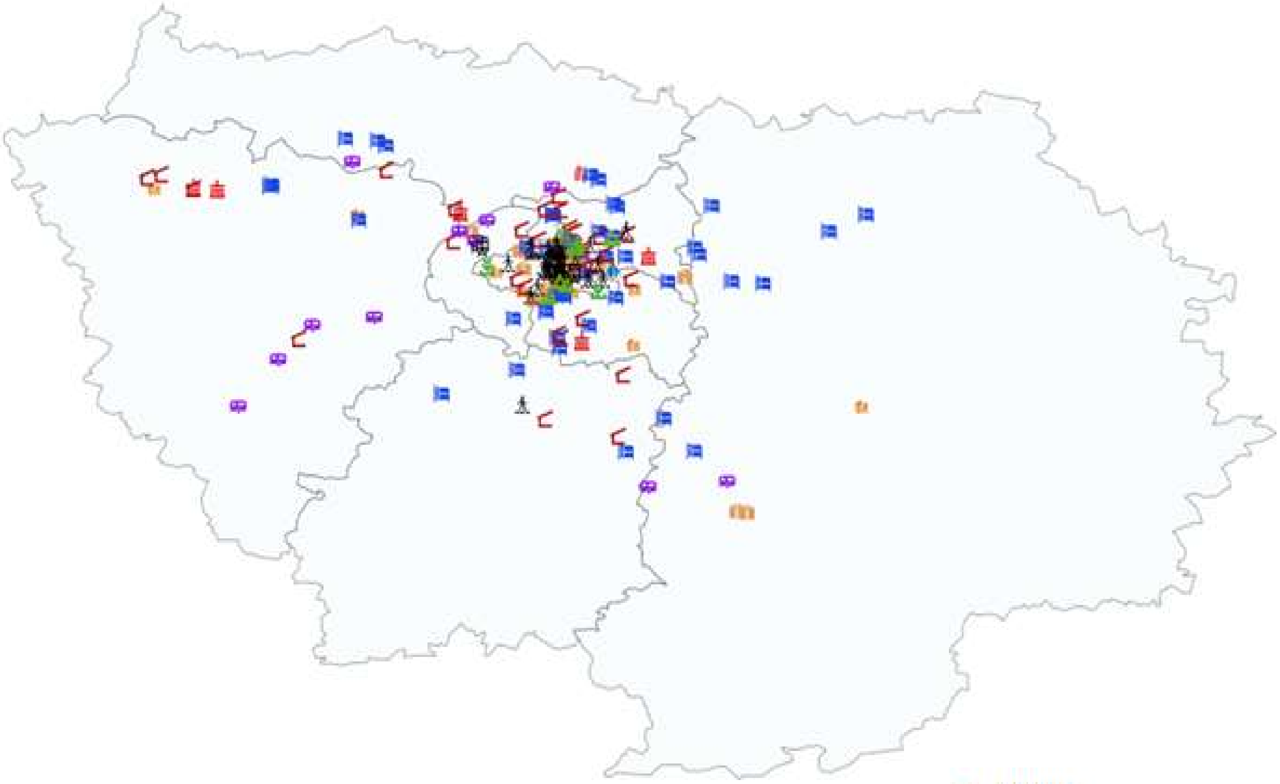

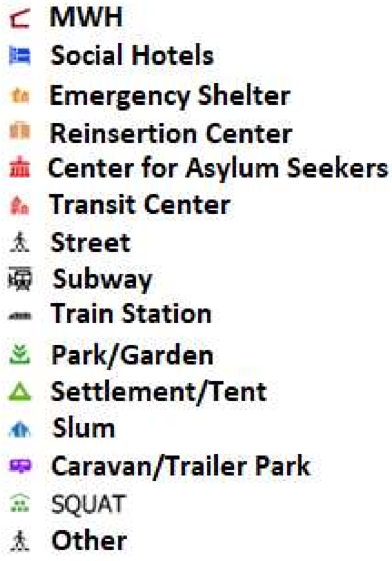


Paris and suburbs

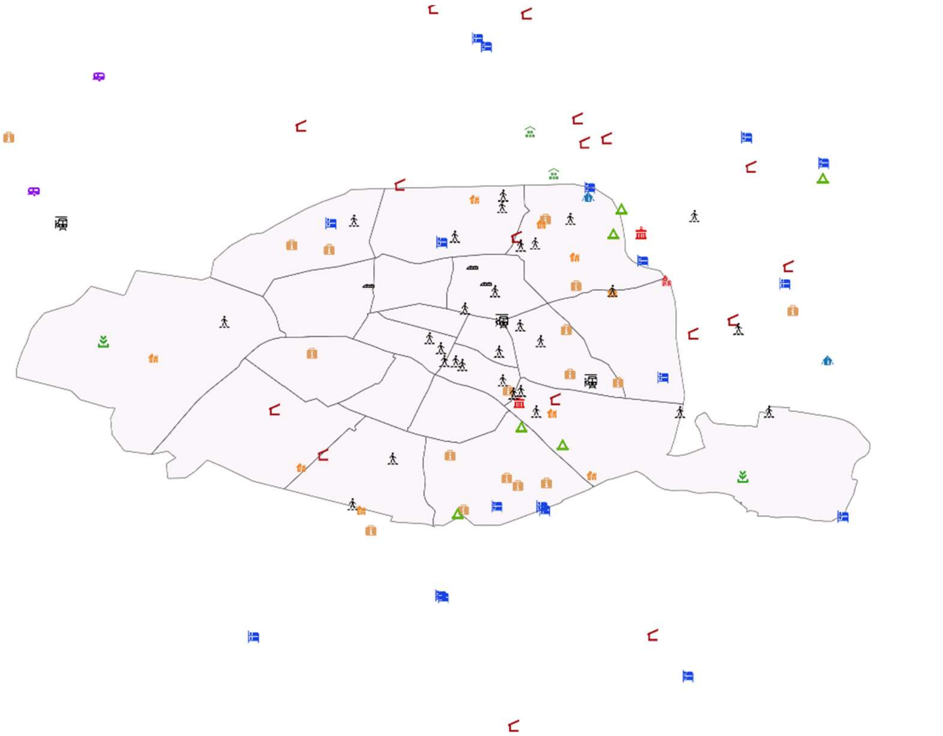

Figure S3. Map of Recruitment Sites in Marseille Metropolitan Area

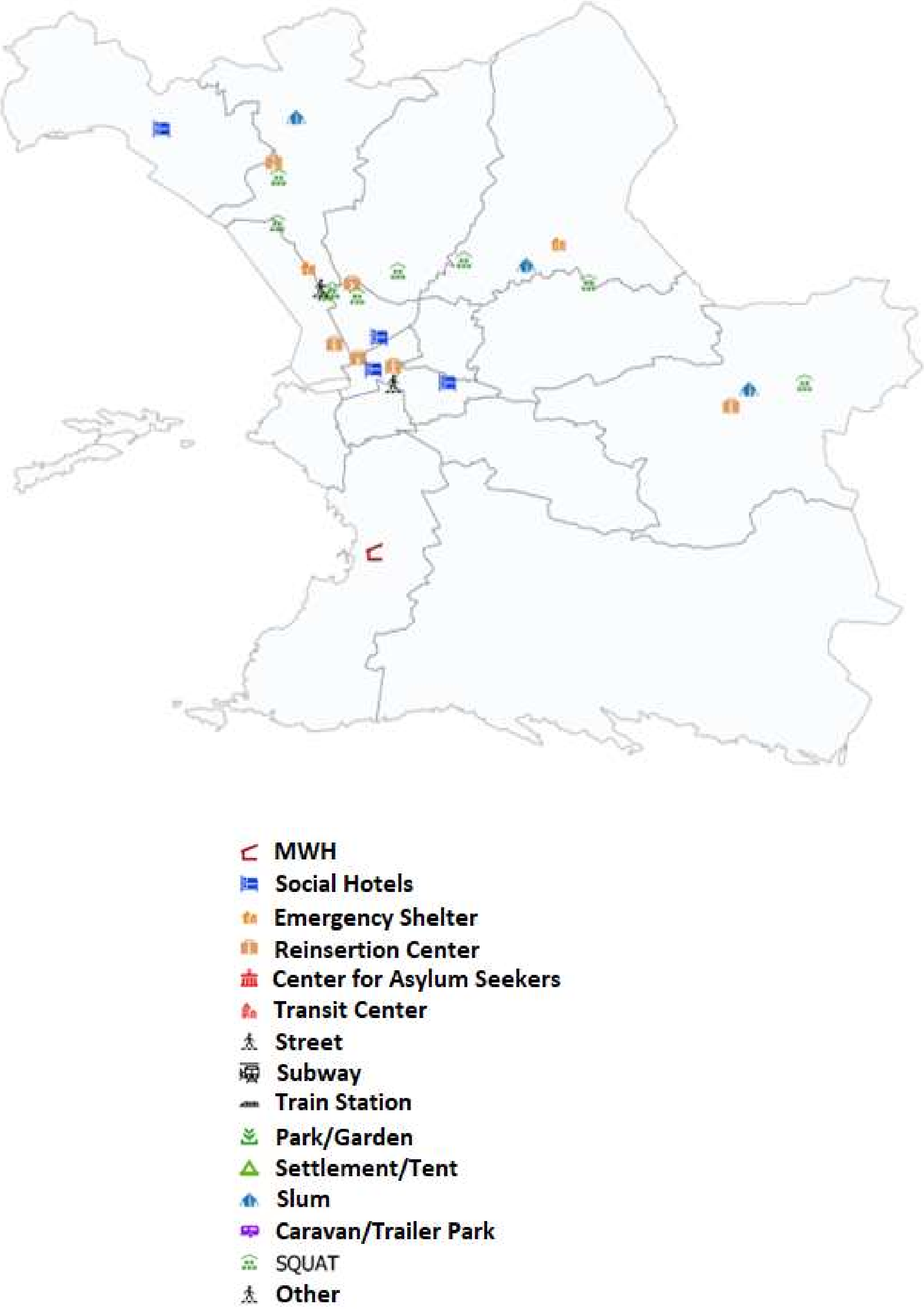

## Figure S4. Flow between original strata and strata for analysis (alluvium)

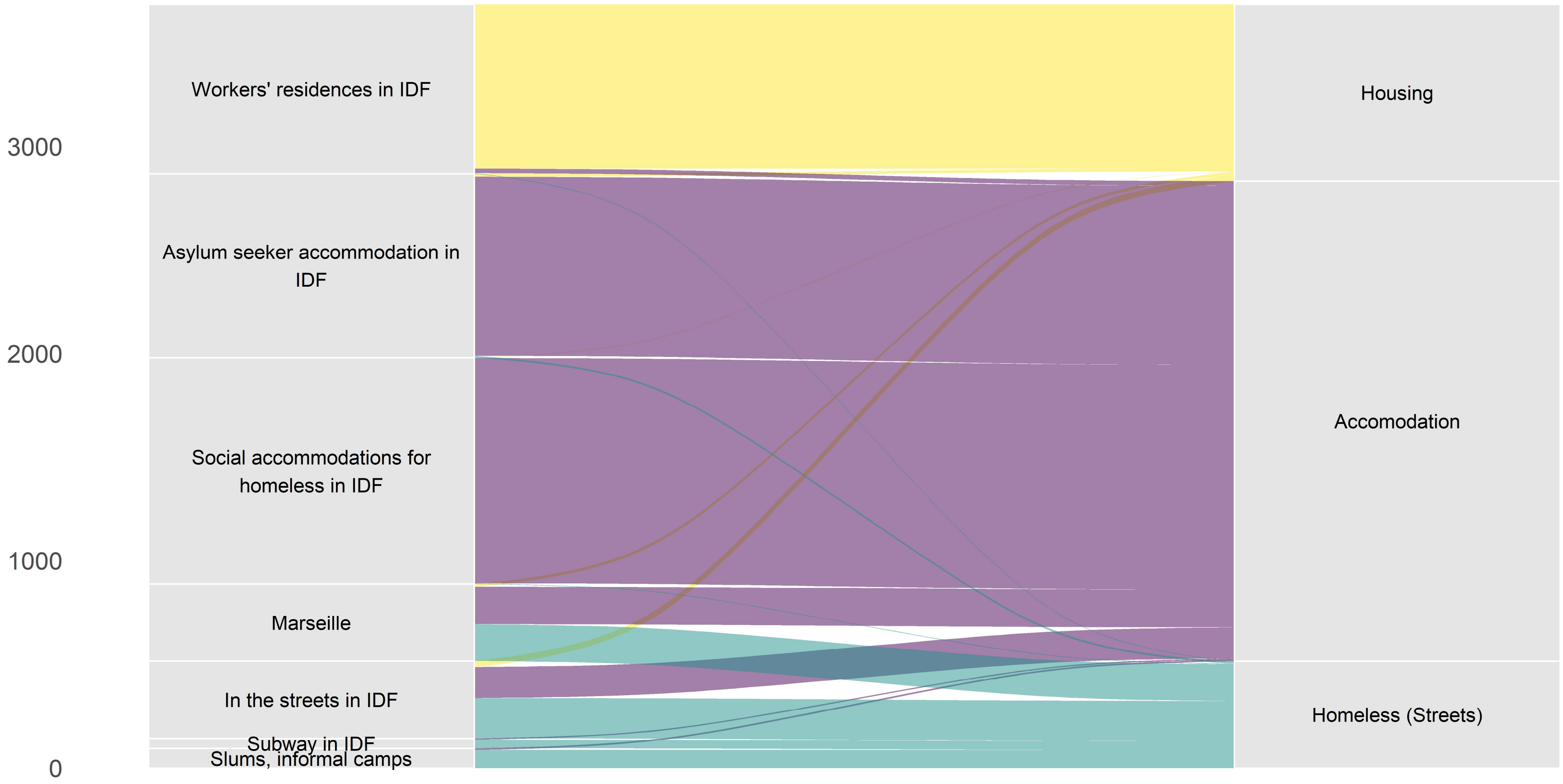


Original recruitment sites (original strata) on the left comumn and new strata for analysis on the right. Each line represents a participant.

## Figure S5. Vaccine Uptake per recruitment site (Weighted Proportions and 95%CI)

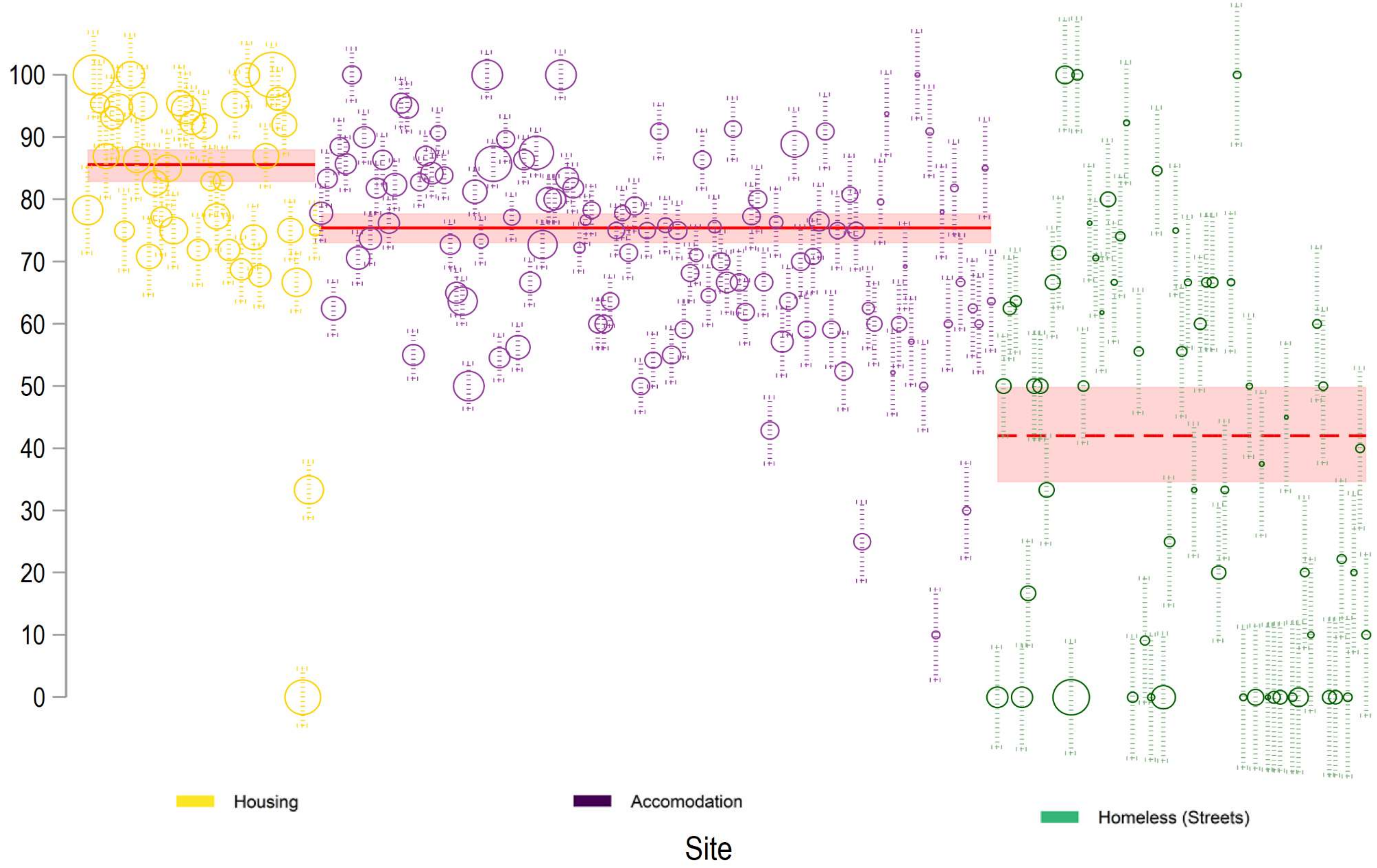

Circles represent proportions of vaccinated individuals per site (size proportional to weight in the sample), vertical dotted lines are the corresponding weighted 95%CI. Red lines are weighted mean for proportion of vaccinated in each strata with their corresponding 95%Cis. Sample sizes are as follows: Weighted N=835/Raw N=855 for Precariously Housed, Weighted N=2,682/Raw N=2,321 for Accommodated and Weighted N=170/Raw N=514 for Streets. Source data are available in Supplementary Data 12

## Figure S6. Forest Plot for the Multivariate Negative Binomial Regression (Site level variables)

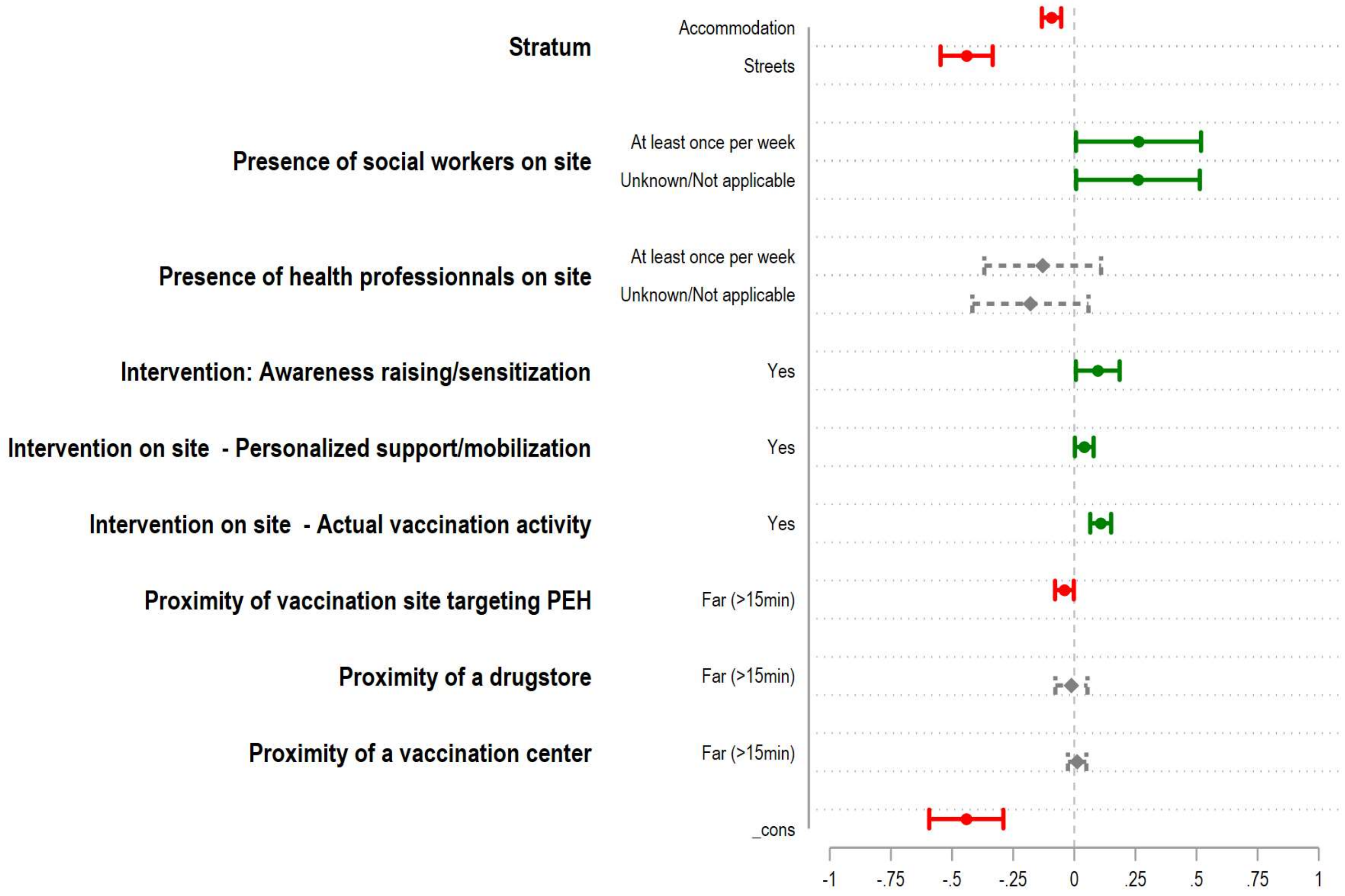

Full dots represent estimates of Adjusted Odds-Ratio and full capped lines their corresponding 95% Confidence Intervals. Red lines for negatively associated factors with total number of vaccinated participants at site-level, green lines for positively associated factors and grey dashed-lines for non-associated factors. Sample size for this model was 3,508. Source data are available in Supplementary Data 8.

## Supplementary References


1. Fondation Abbe Pierre· l'État du mal-logement en France 2021 ( https://www.precarite-energie.org/wp-content/uploads/2022/02/reml2022-web.pdf )
2. Homelessness Services in Europe - EOH Comparative Studies on Homelessness - FEANTSEA 2018 ( https://www.feantsaresearch.org/public/user/Observatory/Feantsa-Studies_08_v02%5B1%5D.pdf )
3. "Journey's End? Old Age in France's Migrant Worker Hostels » by Alistair Hunter , published in 2018 https://doi.org/10.1007/978-3-319-64976-4_1
4. Schwarzinger M, Watson V, Arwidson P, Alla F, Luchini S. (2021). *COVID-19 vaccine hesitancy in a representative working-age population in France: a survey experiment based on vaccine characteristics. Lancet Public Health. avr 2021;6(4):e210-21*
5. Longchamps, C., Ducarroz, S., Crouzet, L., Vignier L. et al. (2021). *COVID-19 vaccine hesitancy among persons living in homeless shelters in France. Vaccine, xxxx.* *https://doi.org/10.1016/j.vaccine.2021.05.012*.
6. 6.Inskip H, Beral V, Fraser P, Haskey J. Methods for age-adjustment of rates. *Stat Med*. 1983;2(4):455-466. doi:10.1002/sim.4780020404
7. Habshah Midi, S.K. Sarkar & Sohel Rana (2010) Collinearity diagnostics of binary logistic regression model, Journal of Interdisciplinary Mathematics, 13:3, 253-267, DOI: 10.1080/09720502.2010.10700699
8. Menard, S. (2000). Coefficients of Determination for Multiple Logistic Regression Analysis. *The American Statistician*, *54*(1), 17–24. https://doi.org/10.2307/2685605
