# Supplementary material for: Estimating COVID-19 vaccine uptake and its drivers among migrants, homeless and precariously housed people in France
Source: Commun Med (Lond). 2023 Feb 20;3:30. doi: 10.1038/s43856-023-00257-1 (PMC9939372; doi:10.1038/s43856-023-00257-1)
Supplement: Supplementary file 15 — Description of Additional Supplementary Files [file 43856_2023_257_MOESM15_ESM.pdf]

Description of Additional Supplementary Files

File Name: Supplementary Data 1.

Description: Data Dictionary for ODK Interview Form

File Name: Supplementary Data 2.

Description: Table 1. Participants characteristics (global and by stratum) – Weighted Proportions and 95% Confidence Intervals

File Name: Supplementary Data 3.

Description: Table S3. Descriptive Analysis (Global and by stratum) – Remaining Variables (weighted analysis)

File Name: Supplementary Data 4.

Description: Table 2. Vaccination characteristics (global and by stratum) - Weighted Proportions and 95% Confidence Intervals

File Name: Supplementary Data 5.

Description: Table S4. Univariate Analysis (unweighted)

File Name: Supplementary Data 6.

Description: Table 3. Drivers of vaccine uptake: multivariable analysis. Final multivel mixed logistic model (after imputation of missing values, unweighted)

File Name: Supplementary Data 7.

Description: Table S5. Description of Site characteristics (unweighted, Site Level)

File Name: Supplementary Data 8.

Description: Table S6. Multivariate Negative Binomial Regression (unweighted, Site-Level)

File Name: Supplementary Data 9.

Description: Table S7. Stratified Multivariate Analysis (unweighted)

File Name: Supplementary Data 10.

Description: Source data for Standardized Vaccination Rates (Figure 2): Excel file containing 3 tabs for each population: France > 18yo, All strata combined and By stratum

File Name: Supplementary Data 11.

Description: Source data for Standardized Vaccination Rates by Age Categories (Figure 3):

Excel file containing 3 tabs for each subcategories: France > 18yo, All strata combined and By stratum

File Name: Supplementary Data 12.

Description: Source data for Vaccine Uptake per recruitment site (Figure S5): Excel file containing 1 tab for data by site
