# Supplementary material for: Estimating COVID-19 vaccine uptake and its drivers among migrants, homeless and precariously housed people in France
Source: Commun Med (Lond). 2023 Feb 20;3:30. doi: 10.1038/s43856-023-00257-1 (PMC9939372; doi:10.1038/s43856-023-00257-1)
Supplement: Supplementary file 16 — Reporting Summary [file 43856_2023_257_MOESM16_ESM.pdf]

nature portfolio

Corresponding author(s): Thomas ROEDERER
NCOMMS-22-28844

Last updated by author(s): 01/20/2023

Reporting Summary

Nature Portfolio wishes to improve the reproducibility of the work that we publish. This form provides structure for consistency and transparency in reporting. For further information on Nature Portfolio policies, see our Editorial Policies and the Editorial Policy Checklist.

Statistics

For all statistical analyses, confirm that the following items are present in the figure legend, table legend, main text, or Methods section.

n/a	Confirmed	
☐	☒	The exact sample size (*n*) for each experimental group/condition, given as a discrete number and unit of measurement
☐	☒	A statement on whether measurements were taken from distinct samples or whether the same sample was measured repeatedly
☐	☒	The statistical test(s) used AND whether they are one- or two-sided<br>*Only common tests should be described solely by name; describe more complex techniques in the Methods section.*
☐	☒	A description of all covariates tested
☐	☒	A description of any assumptions or corrections, such as tests of normality and adjustment for multiple comparisons
☐	☒	A full description of the statistical parameters including central tendency (e.g. means) or other basic estimates (e.g. regression coefficient) AND variation (e.g. standard deviation) or associated estimates of uncertainty (e.g. confidence intervals)
☐	☒	For null hypothesis testing, the test statistic (e.g. *F*, *t*, *r*) with confidence intervals, effect sizes, degrees of freedom and *P* value noted<br>*Give P values as exact values whenever suitable.*
☒	☐	For Bayesian analysis, information on the choice of priors and Markov chain Monte Carlo settings
☒	☐	For hierarchical and complex designs, identification of the appropriate level for tests and full reporting of outcomes
☒	☐	Estimates of effect sizes (e.g. Cohen's *d*, Pearson's *r*), indicating how they were calculated

Our web collection on statistics for biologists contains articles on many of the points above.

Software and code

Policy information about availability of computer code

Data collection	ODK Collect App for Android v1.25.0
Data analysis	Stata v16 and R v3.6.2

For manuscripts utilizing custom algorithms or software that are central to the research but not yet described in published literature, software must be made available to editors and reviewers. We strongly encourage code deposition in a community repository (e.g. GitHub). See the Nature Portfolio guidelines for submitting code & software for further information.

Data

Policy information about availability of data

All manuscripts must include a data availability statement. This statement should provide the following information, where applicable:

- Accession codes, unique identifiers, or web links for publicly available datasets
- A description of any restrictions on data availability
- For clinical datasets or third party data, please ensure that the statement adheres to our policy

The datasets generated during and/or analysed for the current article, alongside the R scripts used for analysis, are available at https://osf.io/gpkum/.
Any exploitation of these public data must be reported to the corresponding author TR.
Readers who wish to use the data for commercial purposes should first contact the corresponding author TR.
The study protocol, detailed statistical analysis plan and electronic forms are available upon request from the corresponding author TR.

Human research participants

Policy information about studies involving human research participants and Sex and Gender in Research.

Reporting on sex and gender	Gender was determined based on self-reporting. Information on individual-data sharing was part of the verbal consent process obtained prior to inclusion in the study. No gender-based analyses was specifically conducted due to lack of power (homeless and migrants are predominantly males) and no prior assumptions could lead to gender-based difference in vaccine uptake. Nonetheless, univariate analyses revealed gender-based differences which finally disappeared in multivariable analyses. We may explore potential gender-specific vaccine uptake drivers in the future.
Population characteristics	Participants aged >18 were recruited at the place they last slept the night. No information on ethnicity was collected (illegal in France). Country of origin, length of stay in France, language, income-generating activities (formal and informal), sources of income, sources of meals, financial and material support received, housing information (over the last 3 months), health-related information (history of COVID-19 infection and/or hospitalization, medical coverage),), support and coping mechanisms (food distribution, support organizations), health literacy and discrimination before/after the COVID-19 crisis. All collected individual information recorded during the interview were priorily described to the participants during informed consent process (as per protocol).
Recruitment	In shelters, migrant workers' hostels, and centres for asylum seekers, individuals were selected using simple random sampling when resident lists or room lists existed, and systematic random sampling otherwise. To ensure the selected person was included, sites were visited repeatedly at different times, including weekends and evenings. If individuals were absent or declined to consent, selected individuals were replaced by another sharing that room or the one adjacent.<br>For people living in the street/camps/slums: All individuals were systematically invited to participate (exhaustive sampling) until stratum sample size was reached. In cases of refusal, the next person apparent was interviewed.<br>In Marseille, a local partner supports an exhaustive cohort of migrants and homeless people. We drew a subsample from this cohort, using simple random sampling, with planned replacements for refusal/absence.
Ethics oversight	The study protocol was approved by the Comité de Protection des Personnes III, Ile de France, Paris on 13 August 2021 (ref. 2021-A01960-41).

Note that full information on the approval of the study protocol must also be provided in the manuscript.

Field-specific reporting

Please select the one below that is the best fit for your research. If you are not sure, read the appropriate sections before making your selection.

☐ Life sciences ☒ Behavioural & social sciences ☐ Ecological, evolutionary & environmental sciences

For a reference copy of the document with all sections, see nature.com/documents/nr-reporting-summary-flat.pdf

Behavioural & social sciences study design

All studies must disclose on these points even when the disclosure is negative.

Study description	Quantitative stratified cross-sectional population-based survey in 2 areas of France
Research sample	Individuals >18 yo spending the night before the interview in the streets, in camps, slums, squats, emergency shelters, social hostels and migrant worker houses in Ile-de-France region and Marseille city. Participants were stratified into 4 groups based on residence/ last night place of stay (following ETHOS typology):<br><br>Streets/camps/slums/squats<br>Social hostels<br>Emergency shelters<br>Centers for asylum seekers<br>Migrant Workers Hostels<br>Marseille cohort of migrants/homeless<br><br>Each stratum was formed to be representative of each population.<br><br>This research sample is of interest for humanitarian actors and non-governmental organizations such as Médecins Sans Frontieres: they were involved heavily in the COVID-19 vaccination campaign amongst homeless, migrants and precariously housed. French Ministry of Health is obviously very concerned with vaccination coverage in those populations. Moreover, vaccination coverage/ vaccine uptake data are rare in such populations, especially real-life estimations.
Sampling strategy	We performed a stratified cross-sectional survey using a two-stage cluster-sampling design.<br><br>Sample Size

We estimated sample size per stratum, based on assumptions reflecting vaccine hesitancy in general French population or people experiencing homelessness reported in the literature. A null hypothesis was made for each stratum (55% or 60%) and sample size was computed considering 80% power (beta), 5% accuracy (bilateral testing) and a 5% type I error (alpha). To account for intra-and inter-class correlations, design effects (DE) were also assumed: a DE of 3 in strata where recruitment sites (first stage) and participants (second stage) were randomly selected , and a DE of 1 in strata where participants were exhaustively offered participation (streets/camps/squats) or sampled from an exhaustive list (Marseille). Number of clusters in each stratum was selected according to logistics and human resources constraints: for ex. a surveyor was expected to interview at least 10 individuals per day in some strata and between 5 and 10 in the Streets stratum. In total, 205 clusters and 3,751 participants were needed.

First Stage (Primary Sampling Units: recruitment sites)

In Ile-de-France, we built sampling frames for each stratum using data provided by various actors involved with these populations; these listed location and size of each site (full list of partners in Appendices). In each stratum, recruitment sites were randomly selected proportionally to their expected size. For people living in the street/camps/slums, we obtained an exhaustive census map recording all homeless and migrants living in subdivisions of Paris in March 2021. Sample by subdivision was proportional to the expected number of individuals living there.
In Marseille, a local partner supports an exhaustive cohort of PEH/PH. We drew a subsample from this cohort, using simple random sampling, with planned replacements for refusal/absence.

Second Stage (Secondary Sampling Units: Individuals)

Sample size per site was calculated in proportion to expected sites population, with participant sampling in the second stage depending on site type.
In shelters, migrant workers' hostels, and centres for asylum seekers, individuals were selected using simple random sampling when resident lists or room lists existed, and systematic random sampling otherwise. To ensure the selected person was included, sites were visited repeatedly at different times, including weekends and evenings. If individuals were absent or declined to consent, selected individuals were replaced by another sharing that room or the one adjacent.
For people living in the street/camps/slums, : All individuals were systematically invited to participate (exhaustive sampling) until stratum sample size was reached. In cases of refusal, the next person apparent was interviewed.

Data collection

Questionnaires were administered by trained interviewers in participant's preferred language. Interviews were conducted in French, English, Arabic, Farsi, Spanish, Turkish, Wolof, and Pulaar directly (many interviewers were speaking at least 2 of those languages). When participant was speaking any another language, a real-time phone translation service (ISM interpretariat: https://ism-interpretariat.fr/interpretariat-par-telephone/) was then used.

Responses were recorded using tablets and the ODK Collect application 1.25.0. COVID-19 vaccine status was verified via the national “Pass sanitaire" phone app (TousAntiCovid) when possible, or paper versions of vaccine certificate otherwise.

Interviewers tried to conduct the interview in a place that respected confidentiality and participant's privacy. When not feasible (eg in the streets, in camps, in congregated settings), the interviewer first explained the survey objectives and interview process to the community and asked non-interviewed individuals to not interfere and allow the interviewee a safe space to answer questions.

Interviewers were aware of study protocol and thus of study hypotheses but were duly trained before field implementation to study procedures such as random sampling, confidentiality and ethics.

Timing

Data were collected between 15 November and 22 December 2021.

Data exclusions

Exclusion criteria were pre-established:

- Language barrier with no in-person or telephone translation available: 33 individuals (time to find phone translater too long)
- Impossibility to give free and informed consent (comprehension barrier, cognitive and/or psychiatric and/or acute addictive disorder, external pressure from family and friends, people under guardianship): 30 individuals, all of them under influence (alcohol or drugs) and/or possibly violent
- Doubt about age (probable minor <18) : 66 without impossibility to verify age (no papers)

Non-participation

Refusal to participate in the study (non-consent) : 2,023 individuals
Main reasons given:
'I don't have the time' (50%)
'I am already vaccinated and I don't see the point of your study' (25%)
'I am not vaccinated and I don't want to answer questions about it' (20%)

Randomization

Randomization was never a plan since stratification was based on residence/place of stay. We collected as many participant information as possible to allow for confounders control in regressions (sociodemographics, social habits, support, COVID-19 related information, opinions on vaccination etc).

Reporting for specific materials, systems and methods

We require information from authors about some types of materials, experimental systems and methods used in many studies. Here, indicate whether each material, system or method listed is relevant to your study. If you are not sure if a list item applies to your research, read the appropriate section before selecting a response.

Materials & experimental systems

n/a	Involved in the study
☒	☐ Antibodies
☒	☐ Eukaryotic cell lines
☒	☐ Palaeontology and archaeology
☒	☐ Animals and other organisms
☒	☐ Clinical data
☒	☐ Dual use research of concern

Methods

n/a	Involved in the study
☒	☐ ChIP-seq
☒	☐ Flow cytometry
☒	☐ MRI-based neuroimaging
